# Physical exercise and hypertension: A retrospective study in southern Sichuan

**DOI:** 10.1097/MD.0000000000037675

**Published:** 2024-04-12

**Authors:** Pei Diao, Kexue Ning, Shaohua Wang, Lijuan He

**Affiliations:** a Department of Physical Education, Neijiang Normal University, Neijiang, Sichuan, China; b College of Agroforestry and Health, The Open University of Sichuan, Chengdu, China; c Cytopathology Department, Department of Pathology, The Affiliated Hospital, Southwest Medical University, Luzhou, Sichuan, China; d Department of Health Management Center, The Affiliated Hospital, Southwest Medical University, Luzhou, Sichuan, China.

**Keywords:** hospital record data, hypertension, lifestyle, physical exercise, retrospective study, risk factors, southern Sichuan

## Abstract

This study aimed to scrutinize the relationship between physical exercise and hypertension, taking into account multiple variables such as age, body mass index (BMI), family history, smoking, and alcohol consumption in the Southern Sichuan population, China, using a retrospective approach based on hospital record data. This retrospective study analyzed data from 946 participants obtained from a hospital electronic medical record system. The data included information regarding participants’ lifestyle factors, family history, and a clinical diagnosis of hypertension. Univariate and multivariate logistic regression models were employed to identify the association between lifestyle factors and hypertension. The study found a hypertension prevalence of 38.5% in the analyzed population. Multivariate analyses identified significant factors associated with hypertension as age (odds ratio [OR]: 1.045, 95% confidence interval [CI]: 1.036–1.054), BMI (OR: 1.107, 95% CI: 1.084–1.132), smoking (OR: 2.299, 95% CI: 1.674–3.157), alcohol consumption (OR: 0.644, 95% CI: 0.478–0.867), and physical exercise (OR: 0.682, 95% CI: 0.506–0.920). Findings from this hospital record-based retrospective study reinforce the multifactorial nature of hypertension. They highlight the significance of physical exercise, along with maintaining optimal BMI and encouraging healthy habits like nonsmoking and moderate alcohol consumption in hypertension prevention. Our findings also underscore the need for future prospective studies to establish causality and explore the generalizability of these results beyond the Southern Sichuan population.

## 1. Introduction

Hypertension, a condition characterized by elevated blood pressure persistently over the normal range, has become a significant public health issue worldwide due to its heightened prevalence and associated risk of cardiovascular diseases.^[1]^ The Chinese adult population has an estimated hypertension prevalence of 23.2%, approximately translating to 245 million individuals.^[2]^

Hypertension complexity stems from its multifactorial etiology, involving an interplay between genetic and environmental factors.^[3]^ Key risk factors implicated in hypertension include advancing age, obesity, family history of hypertension, and lifestyle choices such as smoking and alcohol consumption.^[4–7]^ Recent years have witnessed a growing interest in studying the effect of physical exercise on hypertension.^[8]^

Numerous studies suggest a potential protective role of regular physical exercise in lowering blood pressure and subsequently reducing hypertension risk.^[9–11]^ Furthermore, global health bodies, including the World Health Organization, endorse regular physical activity as a strategy to mitigate hypertension and other non-communicable diseases.^[12]^ However, the degree of influence exerted by physical exercise on hypertension risk varies across different populations and contexts, warranting further exploration.^[13,14]^ Specifically, limited data is available from certain regions like Southern Sichuan, China, leaving a gap in the understanding of physical exercise and hypertension relationship.^[15]^ Therefore, this study aims to delve into the association between physical exercise and hypertension in Southern Sichuan, China, using a retrospective approach based on hospital record data. This study will also examine the potential impacts of age, body mass index (BMI), family history of hypertension, smoking, and alcohol consumption.

Understanding these relationships will be instrumental in devising preventive strategies, particularly in regions with high hypertension prevalence like Southern Sichuan. The findings of this study could potentially inform public health policies and preventive strategies for hypertension, thereby contributing to improved health outcomes in China and globally.^[16]^

## 2. Methods and materials

### 2.1. Study design and population

This retrospective study was conducted to explore the association between physical exercise and hypertension, utilizing medical record data from adults residing in Southern Sichuan, China. We retrieved medical records spanning from January 2019 to December 2021 from local hospital databases. The study included patients aged 18 years and above. Exclusion criteria were set to omit individuals with incomplete data or diagnosed with secondary hypertension, acknowledging that this selection process, while necessary for the integrity of our study, introduces potential selection bias. This bias arises because our sample may not fully represent the broader population, particularly those who do not seek hospital care or who manage their health conditions outside of hospital settings.

### 2.2. Data collection

Data extraction from hospital records focused on demographic information (age and sex), lifestyle factors (physical exercise, smoking, and alcohol consumption), BMI, family history of hypertension, and hypertension diagnosis. Recognizing the potential for information bias in retrospective data collection, we categorized physical exercise into 2 groups to mitigate this issue: regular exercise (engaging in more than 150 minutes of moderate-intensity exercise per week) and irregular/no exercise (engaging in <150 minutes per week). This categorization is based on the recommendations by global health authorities and aims to capture significant lifestyle differences within the population.

### 2.3. Definition of hypertension

Hypertension diagnosis was determined from medical records, adhering to clinical guidelines that define hypertension as a systolic blood pressure of 140 mm Hg or more, diastolic blood pressure of 90 mm Hg or more, or the current use of antihypertensive medication. This standardized definition ensures consistency in identifying hypertensive individuals within our study. However, we recognize that relying on clinical diagnoses may not capture all cases, particularly milder or asymptomatic instances not leading to hospital visits or medication use.

### 2.4. Ethical approval and consent to participate

This investigation was undertaken with the sanction of the Ethics Committee of The Affiliated Hospital of Southwest Medical University (NO:20220811-021) and an exemption for informed consent was obtained from the Ethics Committee of The Affiliated Hospital of Southwest Medical University. All methods were conducted in compliance with relevant guidelines, regulations, and the Declaration of Helsinki.

### 2.5. Statistical analysis

Univariate and multivariate logistic regression analyses were used to explore the relationship between physical exercise and hypertension, adjusting for potential confounders such as age, BMI, smoking, alcohol consumption, and family history of hypertension. Odds ratios (ORs) and 95% confidence intervals (CIs) were computed to estimate the risk of hypertension. All analyses were conducted using the statistical software R version 4.0.2. A *P* value of <.05 was considered statistically significant.

## 3. Results

### 3.1. Demographic and clinical characteristics of the study population

**Figure 1.**
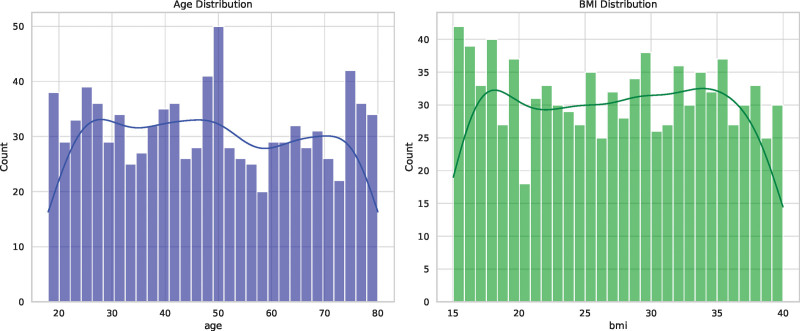
Distribution of age and body mass index (BMI) in the study population. (A) shows the distribution of age among the participants, and (B) depicts the distribution of BMI. The x-axis represents the respective values of age and BMI, while the y-axis indicates the number of participants.

Table 1 summarizes the demographic and clinical characteristics of the study population. The participants’ ages range from 32 to 64 years, with a median age of 48 years. The median BMI among the participants is 27.467, with an interquartile range of 20.893 to 33.613. Regarding family history of hypertension, approximately 37.3% of the participants have a positive family history while 62.7% have no family history. A significant proportion of the participants do not smoke (68.6%), while 31.4% of them are smokers. Alcohol consumption is relatively evenly distributed, with 51.8% of the participants consuming alcohol and 48.2% not consuming alcohol. The majority of the participants (58.9%) engage in regular exercise, while 41.1% of them do not. In terms of hypertension status, 38.5% of the participants have hypertension, whereas 61.5% do not have the condition. The distribution of age and BMI in the study population are shown in Figure 1.

**Table 1:** Demographic and clinical characteristics of the study population.

Characteristics	Overall
Age, median (IQR)	48 (32, 64)
BMI, median (IQR)	27.467 (20.893, 33.613)
Family_history, n (%)	
No	593 (62.7%)
Yes	353 (37.3%)
Smoking, n (%)	
No	649 (68.6%)
Yes	297 (31.4%)
Alcohol, n (%)	
Yes	490 (51.8%)
No	456 (48.2%)
Exercise, n (%)	
No	389 (41.1%)
Yes	557 (58.9%)
Hypertension, n (%)	
No	582 (61.5%)
Yes	364 (38.5%)

All values are presented as median (interquartile range) for continuous variables or number (percentage) for categorical variables. BMI = body mass index. Hypertension status is given as “Yes” (presence of hypertension) and “No” (absence of hypertension).

IQR = interquartile range.

### 3.2. Comparison of demographic and clinical characteristics between hypertensive and non-hypertensive participants

Table 2 illustrates the comparison of demographic and clinical characteristics between hypertensive and non-hypertensive participants. The number of non-hypertensive participants was 582, and the number of hypertensive participants was 364. There were no statistically significant differences between males and females in both groups (*P* = .254). The median age of non-hypertensive and hypertensive participants was 42 and 58 years respectively, and this difference was statistically significant (*P* < .001). Likewise, a significant difference was also observed in the median BMI between the 2 groups (*P* < .001). Family history of hypertension did not significantly differ between the 2 groups (*P* = .280). However, smoking was significantly higher in the hypertensive group (*P* < .001). Similarly, alcohol consumption was also significantly higher in the hypertensive group (*P* = .002). Regarding exercise habits, participants without hypertension were significantly more likely to engage in regular exercise compared to those with hypertension (*P* = .006).

**Table 2:** Comparison of demographic and clinical characteristics between hypertensive and non-hypertensive participants.

Characteristics	No	Yes	*P* value
n	582	364	
Sex, n (%)			.254
Male	302 (31.9%)	175 (18.5%)	
Female	280 (29.6%)	189 (20%)	
Age, median (IQR)	42 (28.25, 57)	58 (42, 69)	<.001
BMI, median (IQR)	25.047 (19.227, 31.336)	31.435 (24.517, 35.505)	<.001
Family_history, n (%)			.280
No	357 (37.7%)	236 (24.9%)	
Yes	225 (23.8%)	128 (13.5%)	
Smoking, n (%)			<.001
No	429 (45.3%)	220 (23.3%)	
Yes	153 (16.2%)	144 (15.2%)	
Alcohol, n (%)			.002
Yes	278 (29.4%)	212 (22.4%)	
No	304 (32.1%)	152 (16.1%)	
Exercise, n (%)			.006
No	219 (23.2%)	170 (18%)	
Yes	363 (38.4%)	194 (20.5%)	

Values are presented as median (interquartile range) for continuous variables or number (percentage) for categorical variables. The *P* values were calculated using Mann–Whitney U test for continuous variables and Chi-square test for categorical variables. A *P* value of <.05 is considered statistically significant.

BMI = body mass index, IQR = interquartile range.

### 3.3. Univariate and multivariate analyses for factors associated with hypertension

**Figure 2.**
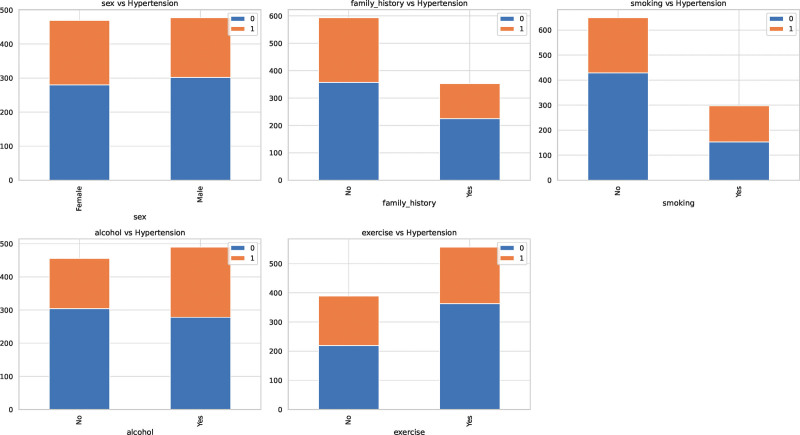
Relationship between categorical variables and hypertension. The stacked bar charts display the distribution of individuals with and without hypertension across different categories of the variables: gender, family history, smoking, alcohol consumption, and exercise. For each variable, the 2 bars represent the 2 categories. The blue segment of the bar signifies the number of individuals without hypertension, while the orange segment indicates those with hypertension.

Table 3 shows the results of the univariate and multivariate analyses to identify factors associated with hypertension. The total number of participants considered in this analysis was 946. In the univariate analysis, age, BMI, smoking, alcohol consumption, and exercise were significantly associated with hypertension. Each year increase in age was associated with a 3.6% higher odds of having hypertension (OR: = 1.036, *P* < .001), and each unit increase in BMI was associated with an 8.7% higher odds (OR: = 1.087, *P* < .001). Participants who smoked had an 83.5% higher odds of having hypertension compared to nonsmokers (OR: = 1.835, *P* < .001). Not consuming alcohol and exercising were associated with lower odds of having hypertension (OR: = 0.656, *P* = .002 and OR = 0.688, *P* = .006 respectively). In the multivariate analysis, where adjustments were made for all the variables in the table, age, BMI, smoking, alcohol consumption, and exercise remained significant. For each year increase in age and each unit increase in BMI, the odds of having hypertension increased by 4.5% (OR: = 1.045, *P* < .001) and 10.7% (OR: = 1.107, *P* < .001) respectively. Participants who smoked were more than twice as likely to have hypertension compared to nonsmokers (OR: = 2.299, *P* < .001). Non-alcohol drinkers and those who exercise regularly had lower odds of having hypertension (OR: = 0.644, *P* = .004 and OR = 0.682, *P* = .012 respectively). The relationship between categorical variables and hypertension is shown in Figure 2.

**Table 3:** Univariate and multivariate analyses for factors associated with hypertension.

Characteristics	Total(N)	Univariate analysis		Multivariate analysis	
		Odds ratio (95% CI)	*P* value	Odds ratio (95% CI)	*P* value
Sex	946				
Male	477	Reference			
Female	469	1.165 (0.896–1.514)	.254		
Age	946	1.036 (1.028–1.044)	**<.001**	1.045 (1.036–1.054)	**<.001**
BMI	946	1.087 (1.067–1.109)	**<.001**	1.107 (1.084–1.132)	**<.001**
Family_history	946				
No	593	Reference			
Yes	353	0.861 (0.655–1.130)	.280		
Smoking	946				
No	649	Reference		Reference	
Yes	297	1.835 (1.388–2.427)	**<.001**	2.299 (1.674–3.157)	**<.001**
Alcohol	946				
Yes	490	Reference		Reference	
No	456	0.656 (0.503–0.854)	**.002**	0.644 (0.478–0.867)	**.004**
Exercise	946				
No	389	Reference		Reference	
Yes	557	0.688 (0.528–0.898)	**.006**	0.682 (0.506–0.920)	**.012**

Bold *P* - values < 0.001 or < 0.05.

Odds ratios (OR) and 95% confidence intervals (CI) are calculated by logistic regression. The *P* values were calculated using univariate and multivariate logistic regression models. In the multivariate model, we adjusted for all variables in the table. A *P* value of <.05 is considered statistically significant.

BMI = body mass index.

## 4. Discussion

In this retrospective study conducted on adults in Southern Sichuan, China, we utilized existing medical records to assess the relationship between hypertension and several factors including age, BMI, smoking, alcohol consumption, exercise, and family history of hypertension. Our analysis revealed a significant association between hypertension and age, BMI, smoking, and alcohol consumption, corroborating with previous research in diverse global contexts.^[17,18]^ Notably, we observed that regular exercise plays a protective role in reducing hypertension risk, which is consistent with the recommendations by the World Health Organization.^[12]^

Our findings underline the significance of age and BMI, 2 well-identified risk factors of hypertension,^[19,20]^ as they were associated with an increased prevalence of hypertension in our study. This suggests a need for targeted interventions towards older individuals and those with higher BMIs as these subgroups appear to be at greater risk.

Another noteworthy risk factor from our analysis is smoking, which has been extensively associated with hypertension and cardiovascular diseases in past literature.^[21,22]^ The finding that alcohol consumption increases the risk of hypertension is also congruent with existing studies, further emphasizing the need for public health initiatives to mitigate smoking and excessive alcohol use.^[23,24]^

Intriguingly, our study supports the growing body of evidence on the protective role of regular exercise against hypertension.^[9,25]^ This finding underscores the potential benefits of advocating for physical activity as part of a comprehensive approach to hypertension control in Southern Sichuan, China, and possibly other similar settings.

However, our study has some limitations. Our study retrospective observational design limits our ability to establish causality between physical exercise and hypertension. Furthermore, the generalizability of our findings may be influenced by the study specific geographical and temporal setting in Southern Sichuan, China. The reliance on hospital records for participant selection and follow-up may not fully capture the spectrum of hypertension severity or the entirety of the population health behaviors. Future research should employ prospective designs and include data from a variety of sources, such as primary care records and community health surveys, to validate our findings and explore the impact of physical exercise on hypertension across different populations and settings.

Our study highlights the association between physical exercise and lower hypertension risk in the Southern Sichuan population, underlining the importance of healthy lifestyle factors in hypertension management. However, the retrospective nature of our study and the reliance on hospital records suggest the need for cautious interpretation of the findings and further research to confirm these associations in broader contexts.

## Acknowledgments

We would like to express our sincere gratitude to all individuals who contributed to the completion of this study. This study followed the EQUATOR network guidelines.

## Authors contributions

**Conceptualization:** Kexue Ning, Shaohua Wang, Lijuan He.

**Data curation:** Pei Diao, Shaohua Wang.

**Formal analysis:** Pei Diao, Shaohua Wang.

**Funding acquisition:** Pei Diao, Lijuan He.

**Investigation:** Kexue Ning.

**Methodology:** Lijuan He.

**Software:** Shaohua Wang, Lijuan He.

**Supervision:** Shaohua Wang.

**Writing – review & editing:** Lijuan He.
